# Supplementary material for: Inflammatory factors and risk of lung adenocarcinoma: a Mendelian randomization study mediated by blood metabolites
Source: Front Endocrinol (Lausanne). 2024 Aug 27;15:1446863. doi: 10.3389/fendo.2024.1446863 (PMC11384989; doi:10.3389/fendo.2024.1446863)
Supplement: Supplementary file 1 [file DataSheet1.zip › supplementary files/S2/GCST90200792/sensitivity-analysis.pdf]

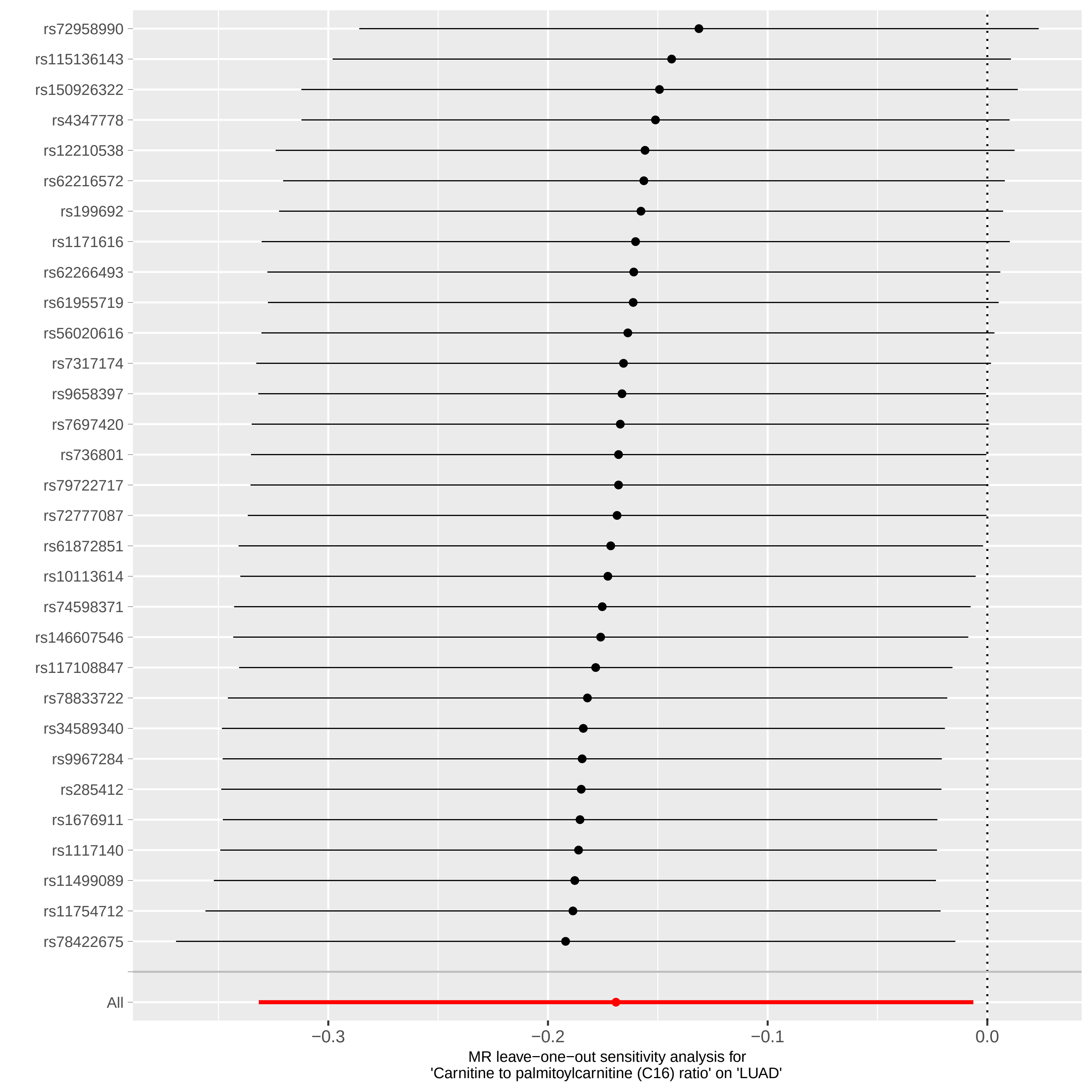
rs72958990
rs115136143
rs150926322
rs4347778
rs12210538
rs62216572
rs199692
rs1171616
rs62266493
rs61955719
rs56020616
rs7317174
rs9658397
rs7697420
rs736801
rs79722717
rs72777087
rs61872851
rs10113614
rs74598371
rs146607546
rs117108847
rs78833722
rs34589340
rs9967284
rs285412
rs1676911
rs1117140
rs11499089
rs11754712
rs78422675
All
−0.3
−0.2
−0.1
0.0
MR leave−one−out sensitivity analysis for
'Carnitine to palmitoylcarnitine (C16) ratio' on 'LUAD'
